# Functional High-Intensity Circuit Training Improves Body Composition, Peak Oxygen Uptake, Strength, and Alters Certain Dimensions of Quality of Life in Overweight Women

**DOI:** 10.3389/fphys.2017.00172

**Published:** 2017-04-03

**Authors:** Billy Sperlich, Birgit Wallmann-Sperlich, Christoph Zinner, Valerie Von Stauffenberg, Helena Losert, Hans-Christer Holmberg

**Affiliations:** ^1^Integrative and Experimental Exercise Science, Institute of Sport Science, University of WürzburgWürzburg, Germany; ^2^Institute of Sport Science, University of WürzburgWürzburg, Germany; ^3^Institute of Health Promotion and Clinical Movement Science, German Sport University CologneCologne, Germany; ^4^School of Kinesiology, University of British ColumbiaVancouver, BC, Canada; ^5^School of Sport Sciences, University of Tromsø—Arctic University of NorwayTromsø, Norway

**Keywords:** aerobic fitness, body composition, female, functional training, interval training, power training

## Abstract

The effects of circuit-like functional high-intensity training (Circuit_HIIT_) alone or in combination with high-volume low-intensity exercise (Circuit_combined_) on selected cardio-respiratory and metabolic parameters, body composition, functional strength and the quality of life of overweight women were compared. In this single-center, two-armed randomized, controlled study, overweight women performed 9-weeks (3 sessions·wk^−1^) of either Circuit_HIIT_ (*n* = 11), or Circuit_combined_ (*n* = 8). Peak oxygen uptake and perception of physical pain were increased to a greater extent (*p* < 0.05) by Circuit_HIIT_, whereas Circuit_combined_ improved perception of general health more (*p* < 0.05). Both interventions lowered body mass, body-mass-index, waist-to-hip ratio, fat mass, and enhanced fat-free mass; decreased ratings of perceived exertion during submaximal treadmill running; improved the numbers of push-ups, burpees, one-legged squats, and 30-s skipping performed, as well as the height of counter-movement jumps; and improved physical and social functioning, role of physical limitations, vitality, role of emotional limitations, and mental health to a similar extent (all *p* < 0.05). Either forms of these multi-stimulating, circuit-like, multiple-joint training can be employed to improve body composition, selected variables of functional strength, and certain dimensions of quality of life in overweight women. However, Circuit_HIIT_ improves peak oxygen uptake to a greater extent, but with more perception of pain, whereas Circuit_combined_ results in better perception of general health.

## Introduction

Programs designed to improve cardiovascular, metabolic, and psychological health have begun to replace low-intensity exercise by repeated short-to-long bouts of high-intensity exercise with intervals of recovery [referred to as high-intensity interval training (HIIT)] (Kessler et al., 2012; Little and Francois, 2014; Elliott et al., 2015; Gielen et al., 2015; Schmitt et al., 2016). In addition to allowing an unlimited number of protocols with different work-to-rest ratios, order of loading and distribution of training intensity, HIIT requires less time than low-intensity-high-volume training, which is attractive, since lack of time appears to be one of the major reasons for not exercising (Godin et al., 1994).

In contrast to the traditional endurance-based HIIT, a relatively novel variation also called functional training/fitness incorporates multi-stimulating, circuit-like, multiple-joint, high-intensity training (Circuit_HIIT_), and becoming of increasing interest to fitness enthusiasts (Buckley et al., 2015). Such exercise more related to strength can improve body composition (Sillanpää et al., 2009; Neves et al., 2017), as well as cardiovascular (Shaw and Shaw, 2009; Ho et al., 2012a,b), metabolic (Schumann et al., 2014; Neves et al., 2017) and functional fitness (Neves et al., 2017) in physically inactive individuals and, in addition, at least in older individuals, certain aspects of quality of life (Sillanpää et al., 2012). However, with increasing exercise intensity, enjoyment declines, and the excessive demands made by HIIT have been proposed to diminish intrinsic motivation and discourage adherence to further exercise (Hardcastle et al., 2014).

Strength exercise such as Circuit_HIIT_, is well known to elevate muscle mass and thereby potentially reduce numerous risk factors for cardiovascular disease (Hurley et al., 1988; Poehlman et al., 2000). In contrast, low-intensity endurance exercise is known to augment plasma volume (Green et al., 1987, 1990), muscular blood flow (Coyle, 1999), and capillary and mitochondrial densities (Hoppeler and Weibel, 2000) thereby improving peak oxygen uptake (Hickson et al., 1981). Improved cardiorespiratory fitness (e.g., maximum oxygen uptake) has been associated with improved health and less premature death (Bouchard et al., 2015). Due to the different responses evoked, functional strength training in the form of Circuit_HIIT_ combined with low-intensity endurance exercise (Circuit_combined_) may lead to synergistic improvements in cardio-respiratory and metabolic parameters, body composition, functional strength and quality of life, which, to the best of our knowledge, has not yet been investigated in detail with respect to overweight women.

Accordingly, we compare here the psycho-physiological responses of physically inactive women performing a multi-stimulating, circuit-like, multiple-joint conditioning program (Circuit_HIIT_), or the same program in combination with low-intensity high-volume exercise (Circuit_combined_). On the basis of the responses discussed above, our hypothesis was that Circuit_HIIT_ improves functional strength and metabolic parameters as well as body composition, but may at the same time impair quality of life, whereas Circuit_combined_ improves cardio-respiratory and metabolic parameters and quality of life.

## Methods

### Participants

In this single-center, two-arm randomized, controlled study, 22 women (age: 23 ± 2 years) recruited via social media platforms and bulletins were initially assigned randomly to perform either Circuit_HIIT_ (*n* = 11) or Circuit_combined;_ (*n* = 8), but three women in the latter group had to withdraw due to time constraints. All were informed in detail about the design of the study, the potential risks and benefits, before providing their written consent to participate. The inclusion criteria were an age of 18–35 years and BMI >25 kg·m^−2^; not having engaged in routine exercise programs for at least 6 months prior to the study; no daily intake of medication; and completion of more than 80% of the training sessions.

Each woman visited the laboratory once before the actual study to become comfortable performing all exercises. All procedures were conducted in accordance with the Declaration of Helsinki and the protocol was pre-approved by the ethical review board of the Sport Science Institute of the University of Würzburg.

#### Overall study design

**Figure 1:**
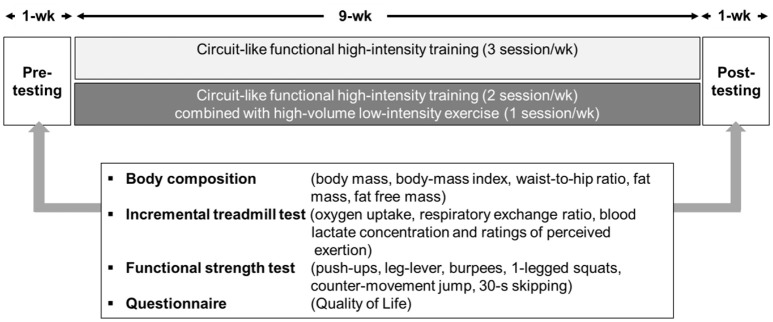
**The timeline and main outcome variables of this study**.

All of the women completed a 9-week intervention involving 3 sessions of either Circuit_HIIT_ or Circuit_combined_ each week. The overall timeline of this intervention is illustrated in Figure 1.

Pre- and post-training testing included assessment of body composition, a treadmill ramp test to assess cardio-respiratory, and metabolic parameters, numerous tests of functional movement, and a questionnaire concerning quality of life. Baseline testing was completed during two visits at least 48 h apart and before assigning the women to one of the interventions. Post-intervention testing, also in two sessions at least 48 h apart, commenced 72 h after the final training session.

### The interventions

The 3 weekly sessions of Circuit_HIIT_ were performed either indoors or outdoors, while Circuit_combined_ involved two sessions of the same nature and a third of low-intensity endurance exercise targeting 65% of peak heart rate (see Table 1 for additional details). Heart rate, especially during the third session during weeks 1, 4, and 7, second session during weeks 2, 5, and 8 and weeks 3, 6 and 9 were monitored continuously (Polar M32, Polar, Oy, Finland).

**Table 1:** **Details of the 9-week Circuit_HIIT_ and Circuit_combined_ training interventions**.

**Week**	**Session**	**Circuit_HIIT_**	**Circuit_combined_**
1, 4, 7	1	6 series of - 30-s burpees + 30-s recovery - 30-s skipping + 30-s recovery - 30-s pull-ups + 30-s recovery - 30-s 1-legged squats + 30-s recovery - 30-s leg levers + 30-s recovery - 30-s push-ups + 30-s recovery 7-min Shuttle run	6 series of - 30-s burpees + 30-s recovery - 30-s skipping + 30-s recovery - 30-s pull-ups + 30-s recovery - 30-s 1-legged squats + 30-s recovery - 30-s leg levers + 30-s recovery - 30-s push-ups + 30-s recovery 7-min Shuttle run
	2	3 series of - 30-s run/burpees +30-s recovery - 30-s burpees + 30-s leg levers - 60-s lunges + 1-min isometric squat - 45-s rows + 1-min recovery - 8x20-s burpees/plank + 10-s recovery	3 series of - 30-s run/burpees +30-s recovery - 30-s burpees + 30-s leg levers - 60-s lunges + 1-min isometric squat - 45-s rows + 1-min recovery - 8x20-s burpees/plank + 10-s recovery
	3	5 series of - 30-s burpees + 30-s recovery - 30-s 1-legged squats + 30-s recovery - 30-s push-ups + 30-s recovery - 30-s crunches + 30-s recovery	4 series of - 10-min jogging + 5-min walking
2, 5, 8	1	3 series of - 45-s lunges/sprints + 30-s recovery - 45-s isometric squat + 30-s recovery - 60-s 1-legged squats + 30-s recovery - 45-s side lunges/sprints + 30-s recovery - 45-s isometric sumo squat + 30-s recovery - 45-s pushups + 30-s rows + 30-s recovery - 30-s plank+30-s skipping + 30-s recovery	3 series of - 45-s lunges/sprints + 30-s recovery - 45-s isometric squat + 30-s recovery - 60-s 1-legged squats + 30-s recovery - 45-s side lunges/sprints + 30-s recovery - 45-s isometric sumo squat + 30-s recovery - 45-s pushups + 30-s rows + 30-s recovery 30-s plank+30-s skipping + 30-s recovery
	2	10 series of - 20-s isometric pull-up/push-up + 40-s run + 30-s recovery - 30-s squats/squat jumps + 30-s run + 30-s recovery 3 series of - 45-s rows + 45-s burpees + 3x 30-s lunges + 30-s run + 30-s recovery - 60-s leg levers + 30-s recovery	4 series of - 10-min jogging + 5-min walking
	3	3 series of - 60-s burpees/run + 30-s recovery - 30-s inchworms/lunges 5 series of - 60-s run + 30-s recovery	10 series of - 20-s isometric pull-up/push-up + 40-s run + 30-s recovery 30-s squats/squat jumps + 30-s run + 30-s recovery 3 series of - 45-s rows + 45-s burpees 3x 30-s lunges + 30-s run + 30-s recovery 60-s leg levers + 30-s recovery
3, 6, 9	1	2 series of - 60-s jump squats + 30-s recovery - 60-s isometric squat + 30-s recovery - 60-s burpees + 30-s recovery - 60-s 1-legged squats + 30-s recovery - 60-s iron mikes + 30-s recovery - 60-s skipping run + 30-s recovery 10-min Shuttle run	2 series of - 60-s jump squats + 30-s recovery - 60-s isometric squat + 30-s recovery - 60-s burpees + 30-s recovery - 60-s 1-legged squats + 30-s recovery - 60-s iron mikes + 30-s recovery - 60-s skipping run + 30-s recovery 10-min Shuttle run
	2	3 series of - 60-s pull-ups + 30-s recovery - 60-s jump rope + 30-s recovery - 60-s push-ups + 30-s recovery - 60-s leg levers + 30-s recovery - 60-s rows + 30-s recovery - 60-s burpees + 30-s recovery 800-m run	3 series of - 20-min jogging + 10-min walking
	3	3 series - 30-s jumping jacks + 30-s recovery - Skipping + 30-s recovery - Squats + 30-s recovery - 3-min burpees + 30-s recovery - 30-s plank + 30-s lunges + 30-s recovery 15-min run	3 series of - 60-s pull-ups + 30-s recovery - 60-s jump rope + 30-s recovery - 60-s push-ups + 30-s recovery - 60-s leg levers + 30-s recovery - 60-s rows + 30-s recovery - 60-s burpees + 30-s recovery 800-m run

#### Anthropometric data and body composition

Height was measured with a folding yardstick with the subjects standing barefoot. Body fat and fat-free mass were assessed to the nearest 0.1 kg with a four-electrode bio-impedance scale (Model 1609N; Tanita Corp, Tokyo, Japan) and body-mass-index (BMI; in kg·m^−2^) then calculated. Since dehydration may affect bio-impedance analysis, we instructed all women to consume 500 mL of water 1 h before measurement. The minimal circumference between the iliac crest and rib cage was designated as the waist circumference, the maximal protuberance of the buttocks as the hip circumference, and the waist-to-hip ratio calculated. In our laboratory setting and with repeated measurements, the error of measurement for the waist and hip circumferences is 1.6 and 1.4 cm, respectively, in line with previous findings (Dhaliwal and Welborn, 2009).

#### The treadmill test

After running for 5 min at 6 km·h^−1^ on a treadmill (Mercury, h/p/Cosmos Sports & Medical GmbH, Nussdorf-Traunstein, Germany), the submaximal heart rate, oxygen uptake, respiratory exchange ratio, blood lactate concentration, and perceived exertion were all assessed. Thereafter, the speed was increased by 1 km·h^−1^ each minute until exhaustion for determination of the peak values of these same parameters (Kuipers et al., 2003). Throughout the testing, oxygen uptake, respiratory exchange ratio and heart rate were monitored with an open circuit breath-by-breath gas and volume analyzer (Metamax 3B, Cortex, Leipzig, Germany), employing standard algorithms to compensate for the delay between oxygen consumption and generation of the signal.

All respiratory data and heart rates were averaged over 30-s intervals. The oxygen uptake at 6 km·h^−1^ was considered to be an indicator of running economy, as described elsewhere (Barnes and Kilding, 2015). The oxygen uptake during the last 30 s of the test was considered to be maximal if (a) this uptake increased <1.0 mL min^−1^ kg^−1^ with elevated power output, (b) the respiratory exchange ratio was >1.10, and/or (c) the heart rate was within 5% of the age-predicted maximum. In all cases, at least two of these three criteria were met.

Both after running for 5 min at 6 km·h^−1^ and at the point of exertion, blood was collected from the right earlobe for analysis of lactate (Lactate Pro 2, Arkray KDK, Kyoto, Japan). With this device and in our laboratory setting, the routinely measured coefficient of variation in repeated measures for blood lactate at rest is 0.07 and 0.04, 0.11, 0.07, 0.06 when running at 70, 80, 90, and 95% of peak heart rate, respectively. At the same time as blood sampling, the rating of perceived exertion (RPE) was assessed employing the 6–20-point Borg scale (Borg, 1970).

#### Testing functional strength

During all tests of functional strength (separated by 3-min intervals of recovery) the women were asked to perform as many push-ups, leg-levers, burpees, and one-legged squats as they could.

### Counter-movement jump and 30-s skipping

The explosive strength of the leg muscles was examined utilizing three counter-movement jumps (OptoJump, MicroGate, Bolzano, Italy) separated by a 1-min rest period, with the best of the three being subjected to further analysis. In the same testing device, the number of times each woman could skip rope within a 30-s period was recorded.

### Assessment of quality of life

Prior to baseline testing and again before post-testing, all of the women completed the German version of the health-related quality of life questionnaire (SF-36), which has been shown to be valid and reliable (Bullinger et al., 1995). This questionnaire assesses general health, physical functioning, mental health, social functioning, vitality, bodily pain, and the roles of physical and emotional limitations, with higher scores (0–100) reflecting better quality of life.

### Statistical analyses

All data were confirmed to be normally distributed by the Kolmogorov-Smirnov test, so that no transformation was required. Repeated-measures ANOVA [time-point (pre- or post-exercise) × group (Circuit_HIIT_ or Circuit_combined_)] was performed for each outcome variable, with an alpha of *p* < 0.05 being considered statistically significant and indicated by ^*^. In addition, the values obtained were evaluated by calculating the effect size partial eta-square ($\eta_{\text{p}}^{2}$). The means and standard deviations (*SD*) for all data sets were calculated and all statistical tests carried out in the SPSS 22.0 software package for Microsoft. The smallest worthwhile effect was defined as the smallest Cohen change in the mean, i.e., 0.2 times the SD of the between-subject baseline values for all participants (Batterham and Hopkins, 2006). Chances of benefit and harm were assessed qualitatively as follows: <1% almost certainly none, 1–5% very unlikely, 5–25% unlikely, 25–75% possibly, 75–95% likely, 95–99% very likely, >99% almost certainly (Hopkins, 2002).

## Results

The women in the Circuit_HIIT_ and Circuit_combined_ groups completed 89 ± 5 and 90 ± 6% of the planned training sessions, respectively. Their pre- and post-values with statistical analysis are documented in Tables 2–**5**.

**Table 2:** **Anthropometric values (means ± SD) for the women in the Circuit_HIIT_ and Circuit_combined_ groups before and after the intervention**.

**Parameter**	**Intervention**	**Before**	**After**	***p*(T)/*p*(T × G)**	**$\eta_{\text{p}}^{2}$(T)/$\eta_{\text{p}}^{2}$(T × G)**	***F***	**Mean effect^a^± 90% CI^b^**	**Qualitative inference**
Body mass [kg]	Circuit_HIIT_	79.7 ± 7.5	77.7 ± 8.2	0.02^*^	0.243	6.41	−2.0 ± 2.1	Likely positive
	Circuit_combined_	83.0 ± 10.5	82.5 ± 11.0	0.121	0.116	2.62		
Body mass index [kg·m^−2^]	Circuit_HIIT_	28.1 ± 2.7	27.3 ± 2.8	0.015^*^	0.263	7.12	−2.0 ± 2.1	Likely positive
	Circuit_combined_	28.3 ± 3.3	28.1 ± 3.2	0.124	0.114	2.58		
Waist-to-hip ratio	Circuit_HIIT_	0.80 ± 0.06	0.75 ± 0.05	0.003^**^	0.359	11.2	−5.1 ± 4.6	Unclear
	Circuit_combined_	0.79 ± 0.03	0.78 ± 0.04	0.340	0.046	0.956		
Fat mass [kg]	Circuit_HIIT_	40.0 ± 4.9	38.1 ± 5.6	0.002^**^	0.401	13.4	−2.5 ± 3.9	Likely positive
	Circuit_combined_	40.2 ± 4.8	39.2 ± 5.6	0.240	0.068	1.47		
Fat free mass [%]	Circuit_HIIT_	26.5 ± 2.4	27.4 ± 2.6	0.003^**^	0.359	11.2	1.6 ± 2.9	Likely positive
	Circuit_combined_	26.4 ± 2.0	26.9 ± 2.5	0.340	0.046	0.956		

*HIIT, high-intensity interval training; p, probability; $\eta_{p}^{\text{2}}$, effect size partial eta-square; T, global effect of time; G, global effect of group; T × G, global effect of Time × Group; F, degrees of freedom*.

* *P < 0.05*,

** *P < 0.01 for the differences between groups*.

a *Refers to Circuit_HIIT_ minus Circuit_combined_*.

b *± 90% CI: add or subtract this number from the mean effect to obtain the 90% confidence intervals for the true difference*.

Body mass, body-mass index, waist-to-hip ratio and fat mass declined and fat-free mass increased to the same extent in both groups (Table 2).

Ratings of perceived exertion following either intervention were lower than at baseline. Peak oxygen uptake improved to a greater extent following Circuit_HIIT_ than Circuit_combined_ (Table 3).

**Table 3:** **Cardio-respiratory and metabolic values and perceived exertion (means ± SD) of the women in the Circuit_HIIT_ and Circuit_combined_ groups before and after the intervention**.

	**Intervention**	**Before**	**After**	***p*(T)/*p*(T × G)**	**$\eta_{\text{p}}^{2}$(T)/$\eta_{\text{p}}^{2}$(T × G)**	***F***	**Mean effect^a^± 90% CI^b^**	**Qualitative inference**
**VALUES AT A SUBMAXIMAL RUNNING SPEED OF 6 KM·H^−1^**								
Heart rate [bpm]	Circuit_HIIT_	166 ± 18	160 ± 18	0.087	0.140	3.250	−2.3 ± 4.4	Likely positive
	Circuit_combined_	167 ± 14	165 ± 12	0.424	0.032	0.665		
Oxygen uptake [ml·min^−1^]	Circuit_HIIT_	2,070 ± 206	2,110 ± 141	0.742	0.006	0.112	3.4 ± 6.8	Likely positive
	Circuit_combined_	2,130 ± 311	2,110 ± 304	0.391	0.039	0.770		
Respiratory exchange ratio	Circuit_HIIT_	0.93 ± 0.09	0.91 ± 0.10	0.136	0.113	2.430	2.5 ± 8.3	Unclear
	Circuit_combined_	0.94 ± 0.10	0.89 ± 0.07	0.556	0.019	0.359		
Blood lactate concentration [mmol·L^−1^]	Circuit_HIIT_	4.4 ± 2.0	3.1 ± 1.2	0.017	0.265	6.850	−25.3 ± 17.7	Very likely positive
	Circuit_combined_	4.0 ± 2.3	3.7 ± 1.7	0.088	0.146	3.25		
Ratings of perceived exertion [a.u.]	Circuit_HIIT_	12.0 ± 1.6	9.5 ± 2.0	<0.001^***^	0.683	36.6	−7.6 ± 11.5	Likely positive
	Circuit_combined_	13.1 ± 2.0	11.1 ± 1.9	0.510	0.026	0.452		
**PEAK VALUES AT THE POINT OF EXERTION**								
Maximal oxygen uptake [ml·min^−1^]	Circuit_HIIT_	2,630 ± 297	2,900 ± 298	<0.001^***^	0.612	30.0	5.8 ± 4.9	Very likely positive
	Circuit_combined_	2,560 ± 348	2,670 ± 398	0.038^*^	0.207	4.95		
Respiratory exchange ratio	Circuit_HIIT_	1.23 ± 0.08	1.17 ± 0.08	0.102	0.134	2.95	−2.4 ± 7.6	Unclear
	Circuit_combined_	1.18 ± 0.09	1.15 ± 0.07	0.591	0.016	0.299		
Blood lactate concentration [mmol·L^−1^]	Circuit_HIIT_	7.0 ± 2.1	8.0 ± 2.8	0.645	0.011	0.220	26.5 ± 37.4	Likely positive
	Circuit_combined_	7.0 ± 2.2	6.5 ± 2.5	0.128	0.118	2.54		
Ratings of perceived exertion [a.u.]	Circuit_HIIT_	18.2 ± 1.5	17.9 ± 2.2	0.396	0.038	0.755	0.9 ± 9.2	Unclear
	Circuit_combined_	17.8 ± 1.8	17.3 ± 1.6	0.801	0.003	0.065		

*HIIT, high-intensity interval training; p, probability; $\eta_{p}^{\text{2}}$, effect size partial eta-square; T, global effect of time; G, global effect of group; T × G, global effect of Time × Group; F, degrees of freedom; a.u, arbitrary units*.

* *P < 0.05*,

*** *P < 0.001 for the differences between groups*.

a *Refers to Circuit_HIIT_ minus Circuit_combined_*.

b *± 90% CI: add or subtract this number from the mean effect to obtain the 90% confidence intervals for the true difference*.

The number of push-ups, burpees, one-legged squats and 30-s skipping, as well as the counter-movement jumping height improved after both Circuit_HIIT_ and Circuit_combined_, with no differences between these groups (Table 3). The number of leg-levers was improved to a greater extent by Circuit_HIIT_ than Circuit_combined_ (Table 4).

**Table 4:** **Funtional performance (means ± *SD*) of the women in the Circuit_HIIT_ and Circuit_combined_ groups before and after the intervention**.

**Parameter**	**Intervention**	**Before**	**After**	***p*(T)/*p*(T × G)**	**$\eta_{\text{p}}^{2}$(T)/$\eta_{\text{p}}^{2}$(T × G)**	***F***	**Mean effect^a^ ± 90% CI^b^**	**Qualitative inference**
Push-ups [n]	Circuit_HIIT_	7.5 ± 3.6	19.6 ± 4.2	<0.001^***^	0.869	119	−23.2 ± 47.8	Unclear
	Circuit_combined_	4.3 ± 4.0	17.4 ± 6.1	0.663	0.011	0.196		
Leg-levers [n]	Circuit_HIIT_	15.7 ± 11.0	58.5 ± 22.3	<0.001^***^	0.754	55.0	120.4 ±84.5	Likely positive
	Circuit_combined_	15.9 ± 8.0	28.3 ± 6.5	0.001^***^	0.480	16.6		
Burpees [n]	Circuit_HIIT_	9.2 ± 3.7	24.9 ± 9.9	<0.001^***^	0.753	57.8	−28.0 ± 33.6	Likely negative
	Circuit_combined_	7.0 ± 2.7	27.6 ± 10.8	0.321	0.052	1.04		
1-Legged squats [n]	Circuit_HIIT_	18.0 ± 8.2	36.9 ± 10.1	<0.001^***^	0.868	125	−32.7 ± 24.6	Very likely negative
	Circuit_combined_	13.7 ± 5.9	39.9 ± 9.4	0.086	0.147	3.28		
Counter-movement jump [cm]	Circuit_HIIT_	23.8 ± 7.0	28.3 ± 7.2	0.017^*^	0.253	6.79	16.4 ±16.8	Likely positive
	Circuit_combined_	24.8 ± 5.1	25.3 ± 3.2	0.050	0.178	4.33		
30-s Skipping [n]	Circuit_HIIT_	84.0 ± 32.2	116 ± 8.3	<0.001^***^	0.679	38.0	−19.0 ± 25.1	Likely negative
	Circuit_combined_	76.6 ± 21.9	136 ± 37.0	0.074	0.167	3.60		

*HIIT, high-intensity interval training; p, probability; $\eta_{p}^{\text{2}}$, effect size partial eta-square; T, global effect of time; G, global effect of group; T × G, global effect of Time × Group; F, degrees of freedom*.

* *P < 0.05*,

*** *P < 0.001 for the differences between groups*.

a *Refers to the Circuit_HIIT_ minus Circuit_combined_*.

b *± 90% CI: add or subtract this number from the mean effect to obtain the 90% confidence intervals for the true difference*.

Physical and social functioning, vitality, role of emotional limitations and mental health improved following both Circuit_HIIT_ and Circuit_combined_ (Table 5). However, perception of physical pain was higher only after Circuit_HIIT_ and perception of general health was enhanced only by Circuit_combined_.

**Table 5:** **Quality of Life (arbitrary units, means ± SD) for the women in the Circuit_HIIT_ and Circuit_combined_ groups before and after the intervention**.

**Dimension**	**Intervention**	**Before**	**After**	***p*(T)/*p*(T × G)**	**$\eta_{\text{p}}^{2}$(T)/$\eta_{\text{p}}^{2}$(T × G)**	***F***	**Mean effect^a^ ± 90% CI^b^**	**Qualitative inference**
Physical functioning	Circuit_HIIT_	94 ± 5.5	98 ± 3	0.008^**^	0.317	8.82	−7.6 ± 10.4	Likely negative
	Circuit_combined_	87 ± 14	97 ± 4	0.228	0.075	1.55		
Role of physical limitations	Circuit_HIIT_	100 ± 0	98 ± 8	0.439	0.032	0.624	0.3 ± 16.1	Unclear
	Circuit_combined_	95 ± 16	93 ± 17	0.970	0.000	0.001		
Pain	Circuit_HIIT_	96 ± 9	74 ± 19	0.008^**^	0.320	8.93	−24.5 ± 14.3	Very likely negative
	Circuit_combined_	91 ± 19	90 ± 17	0.016^*^	0.270	7.04		
Perception of general health	Circuit_HIIT_	74 ± 15	74 ± 18	0.039^*^	0.206	4.94	−23.1 ± 16.3	Very likely negative
	Circuit_combined_	66 ± 18	83 ± 17	0.037^*^	0.210	5.05		
Vitality	Circuit_HIIT_	58 ± 13	66 ± 10	0.001^**^	0.427	14.2	−11.7 ± 17.4	Likely negative
	Circuit_combined_	49 ± 14	63 ± 13	0.336	0.049	0.975		
Social functioning	Circuit_HIIT_	77 ± 18	94 ± 10	0.009^**^	0.306	8.38	18.0 ± 19.8	Likely positive
	Circuit_combined_	91 ± 10	96 ± 8	0.130	0.116	2.50		
Role of emotional limitations	Circuit_HIIT_	73 ± 29	91 ± 22	0.015^*^	0.273	7.14	3.0 ± 42.0	Unclear
	Circuit_combined_	77 ± 39	93 ± 21	0.909	0.001	0.014		
Mental health	Circuit_HIIT_	72 ± 14	76 ± 11	0.013^*^	0.285	7.57	−8.0 ± 12.4	Likely negative
	Circuit_combined_	68 ± 11	79 ± 12	0.222	0.078	1.60		

*HIIT, high-intensity interval training; p, probability; $\eta_{p}^{\text{2}}$, effect size partial eta-square; T, global effect of time; G, global effect of group; T × G, global effect of Time × Group; F, degrees of freedom*.

* *P < 0.05*,

** *P < 0.01 for the difference between groups*.

a *Refers to Circuit_HIIT_ minus Circuit_combined_*.

b *± 90% CI: add or subtract this number from the mean effect to obtain the 90% confidence intervals for the true difference*.

## Discussion

The major findings of this comparison of several psycho-physiological responses of overweight women to 9 weeks of either Circuit_HIIT_ or Circuit_combined_ were as follows:

1. With Circuit_HIIT_, perception of physical pain and peak oxygen uptake both rose to a greater extent.
2. Perception of general health was improved more by Circuit_combined_.
3. Both interventions caused the following changes to a similar degree:
  - lowering of body mass, body-mass index, waist-to-hip ratio, and fat mass and increase in fat-free mass;
  - decreased ratings of perceived exertion during submaximal treadmill running;
  - improvements in the number of push-ups, burpees, one-legged squats, and 30-s skipping, as well as counter-movement jump height;
  - improvements in physical and social functioning, role of physical limitations, vitality, role of emotional limitations and mental health.

Numerous investigations have focused on the effect of HIIT on parameters of cardio-respiratory fitness including VO_2peak_ (Stoggl and Sperlich, 2014; Schmitt et al., 2016; Zinner et al., 2016). Since this value limits ATP production via oxidative phosphorylation and is thus strongly associated with cardiorespiratory fitness, as well as general health and premature death, it is frequently utilized as an integrative indicator of cardiopulmonary fitness (Bassett and Howley, 2000; Bouchard et al., 2015). Here, peak oxygen uptake was improved after 9 weeks with 3 weekly sessions of either Circuit_HIIT_ (10.1%) or Circuit_combined_ (4.4%). The overall greater response of VO_2peak_ to high-intensity exercise can be explained by better central adaptation, including augmented plasma and blood volumes (Convertino et al., 1980; Green et al., 1987; Graham et al., 2016) with elevated stroke volume (Green et al., 1990; Goodman et al., 2005).

From this point of view, we may conclude that three sessions of Circuit_HIIT_ enhance VO_2peak_ more effectively than two sessions of Circuit_HIIT_ and one session of low-intensity exercise (Circuit_combined_).

The Circuit_HIIT_ participants performed primarily resistance training (i.e., squats, lunges, push-ups, etc.) in combination with repeated sprints, with very little rest between these exercises. Although this regimen was more related to strength, the women enhanced their cardiorespiratory fitness (i.e., oxygen uptake) to an extent similar to that observed with earlier circuit-based exercise (Gettman et al., 1982; Beckham and Earnest, 2000). A 7–12% increase in peak oxygen uptake following 4–12 weeks of body weight circuit training is consistent with previous reports (Gettman et al., 1982; McRae et al., 2012). However, our present observations reveal that 3 weekly sessions of Circuit_HIIT_ improve peak oxygen uptake in a time-effective manner.

Circuit_combined_ consisted of two of the weekly Circuit_HIIT_ sessions and an additional weekly session of high-volume exercise (60–105 min at approximately 65% of peak oxygen uptake). In the case of well-trained endurance athletes a combination of HIIT and high-volume low-intensity exercise might be more beneficial than either alone, since a) low-intensity endurance exercise (<2 mM blood lactate or ~55–85% peak heart rate) allows the body to recover sufficiently and b) excess HIIT may exert a negative impact on the autonomic nervous system (Chwalbinska-Moneta et al., 1998; Esteve-Lanao et al., 2007). Although the combination of low-volume, high-intensity exercise and high-volume, low-intensity exercise (so-called polarized intensity distribution) led to greater improvements in selected endurance values for elite endurance athletes (Stöggl and Sperlich, 2015), our present findings indicate that this is not the case for overweight women performing multi-stimulating, circuit-like, multiple-joint training together with high-volume low-intensity exercise (Circuit_combined_).

In the current investigation, the women in the Circuit_HIIT_ group perceived more pain, with no change in perception of general health. High-intensity strength training with eccentric components such as those involved in multi-stimulating, circuit-like, multiple-joint training induces muscle soreness. Although in general in adults, perception of muscle pain is attenuated as training proceeds, (Levinger et al., 2007), the women in the Circuit_HIIT_ group perceived more pain after the 9-week intervention. The injury rate associated with multi-stimulating, circuit-like, multiple-joint training is approximately 20% (Weisenthal et al., 2014), but none of the women in either of our groups mentioned any injury. However, elevated pain in connection with HIIT may discourage untrained individuals from performing regular physical activity (Hardcastle et al., 2014; Del Vecchio et al., 2015), although previous studies showed no effect of high-intensity exercise on perceived pain in female patients (Schmitt et al., 2016). Further studies are needed to clarify the effect of high-intensity training, especially short- and long-term, on perceived pain. However, Circuit_HIIT_ and Circuit_combined_ induced improvements in several dimensions of quality of life, which are perhaps the most important reasons to continue training. To summarize, in the present investigation Circuit_combined_ appears to have augmented certain aspects of quality of life somewhat more, because there was no enhanced perception of pain.

### Limitations

This study was not designed specifically to assess improvements in body composition, but rather to compare the responses of cardio-respiratory and metabolic parameters, body composition, functional strength and quality of life to Circuit_HIIT_ and Circuit_Combined_. Although we instructed our participants not to alter their eating habits, we cannot exclude the possibility that some nonetheless altered their nutritional intake. Since the study was relatively time-consuming, we did not ask the women to maintain nutritional diaries as well.

Since Circuit_HIIT_ and Circuit_Combined_ can be designed in very many different ways, we cannot be certain that the exercise described here is superior to other protocols. Nor can we generalize the responses observed to other populations, such as men, younger, and older individuals, or those with greater fitness.

### Practical implications

Depending on the training goal, both Circuit_HIIT_ and Circuit_combined_ achieve potent improvements in body composition, selected parameters of functional strength, and certain dimensions of quality of life in overweight women. Moreover, Circuit_HIIT_ improves peak oxygen uptake, which is associated with improved health and less risk for premature death (Bouchard et al., 2015), in less training time, but with more perception of pain. More pain could, in turn, diminish pleasure and, thereby, the motivation to continue a program of exercise (Hardcastle et al., 2014).

Circuit_combined_ appears to improve perception of general health to a greater extent than Circuit_HIIT_ and, if this is the goal, Circuit_combined_ seems to be the method of choice.

## Conclusion

Nine weeks of multi-stimulating, circuit-like, multiple-joint training involving either Circuit_HIIT_ or Circuit_combined_ improved the body composition, selected variables of functional strength and certain dimensions of quality of life in overweight women. Circuit_HIIT_ also improved peak oxygen uptake, but with more perception of pain. Circuit_combined_ appears to improve perception of general health to a greater extent than Circuit_HIIT_.

## Author contributions

BS, BWS, CZ, VVS, HL, HCH designed and approved the methods, analyzed data, and assisted in manuscript writting. BS, VVS, HL, CZ performed data collection.

### Conflict of interest statement

The authors declare that the research was conducted in the absence of any commercial or financial relationships that could be construed as a potential conflict of interest.
